# Why are physicians not persuaded by scientific evidence? A grounded theory interview study

**DOI:** 10.1186/1472-6963-6-92

**Published:** 2006-07-27

**Authors:** Miho Sekimoto, Yuichi Imanaka, Nobuko Kitano, Tatsuro Ishizaki, Osamu Takahashi

**Affiliations:** 1 Department of Healthcare Economics and Quality Management, Kyoto University Graduate School of Medicine, Yoshida Konoe-cho, Sakyo-ku, Kyoto, 606-8501, Japan; 2 Department of General Medicine and Clinical Epidemiology, Kyoto University Graduate School of Medicine, 54 Kawahara-cho, Shogoin, Sakyo-ku, Kyoto, 606-8507, Japan

## Abstract

**Background:**

The government-led "evidence-based guidelines for cataract treatment" labelled pirenoxine and glutathione eye drops, which have been regarded as the standard care for cataracts in Japan, as lacking evidence of effectiveness, causing great upset among ophthalmologists and professional ophthalmology societies. This study investigated the reasons why such "scientific evidence of treatment effectiveness" is not easily accepted by physicians, and thus, why they do not change their clinical practices to reflect such evidence.

**Methods:**

We conducted a qualitative study based on grounded theory to explore physicians' awareness of "scientific evidence" and evidence-supported treatment in relation to pirenoxine and glutathione eye drops, and to identify current barriers to the implementation of evidence-based policies in clinical practice. Interviews were conducted with 35 ophthalmologists and 3 general practitioners on their prescribing behaviours, perceptions of eye drop effectiveness, attitudes toward the eye drop guideline recommendations, and their perceptions of "scientific evidence."

**Results:**

Although few physicians believed that eye drops are remarkably effective, the majority of participants reported that they prescribed eye drops to patients who asked for them, and that such patients accounted for a considerable proportion of those with cataracts. Physicians seldom attempted to explain to patients the limitations of effectiveness or to encourage them to stop taking the eye drops. Physicians also acknowledged the benefits of prescribing such drugs, which ultimately outweighed any uncertainty of their effectiveness. These benefits included economic incentives and a desire to be appreciated by patients. Changes in clinical practice were considered to bring little benefit to physicians or patients. Government approval, rarity of side effects, and low cost of the drops also encouraged prescription.

**Conclusion:**

Physicians occasionally provide treatment without expecting remarkable therapeutic effectiveness, as exemplified by the use of eye drops. This finding highlights that scientific evidence alone cannot easily change physicians' clinical practices, unless evidence-based practices are accepted by the general public and supported by health policy.

## Background

Evidence-based Medicine (EBM) is increasingly attracting worldwide attention in public policy [1], resulting in the mass production of evidence-based clinical guidelines [2,3]. The paradigm of EBM was introduced to Japan in the mid-1990s and has quickly gained immense popularity among healthcare professionals. The Japanese government has organized ad hoc groups to develop evidence-based, clinical guidelines targeting 23 high priority diseases. However, recent opinions tend to be critical of EBM, arguing that it has not been adequately integrated into Japanese medical practices [4]. Some evidence-based guidelines have come under attack, including those established for cataract treatment. Released in 2003, the guidelines pointed out that no scientific evidence supports the effectiveness of pirenoxine and glutathione eye drops, which are commonly prescribed to treat the condition. Several newspapers reported that healthcare providers are prescribing ineffective eye drops in order to increase the number of patient consultations. Understandably, this media coverage caused much distress among ophthalmologists and professional ophthalmology societies in Japan [5].

Although EBM advocates argue that misunderstandings surrounding EBM have created this situation, it is also important to understand the dynamics that govern physicians' acceptance or rejection of evidence-based clinical guidelines. We investigated physicians' attitudes and opinions regarding the Evidence-based Clinical Guidelines for Cataract Treatment, and attempted to identify the reasons why physicians sometimes conduct clinical practices that are not supported by scientific evidence. This study focused on physicians' prescribing behaviours, perceptions of pirenoxine and glutathione eye drop effectiveness, attitudes toward the guideline's recommendations, and attitudes toward "scientific evidence." Pirenoxine and glutathione eye drops are commonly prescribed to an estimated 1.3 million cataract patients in Japan. This study also investigated why the guidelines were so controversial among Japanese ophthalmologists, and whether they have influenced physicians' practices. Such exploration is anticipated to reveal the reasons why evidence-based practice has been difficult to integrate into clinical practices in Japan, as well as in other countries.

## Methods

### Background data regarding the use of pirenoxine and glutathione eye drops

Pirenoxine eye drops were first approved by the government in 1958 as a preventive drug for the initial stage of senile cataract. This approval was based on three studies [6-8], which used animal experiments and patho-physiological principles to conclude that eye drops have a preventative effect against senile cataract. The evidence-based guidelines reviewed data relevant to the treatment of cataract, recommending surgery as the definitive treatment and determining the effectiveness of eye drops to be uncertain (See Appendix). Based on a systematic literature review, the guidelines identified three clinical trials conducted in Japan that investigated the effectiveness of pirenoxine and glutathione eye drops, and concluded that neither demonstrated effectiveness [9-11]. Although these clinical trials reported that lens opacity was better in the eye drop group compared to its control, this assessment was based on a subjective measurement. There were no double-blind studies to evaluate the eye drops in terms of visual acuity and no adverse effects were reported in the trials.

### Participants and methods

This study used Grounded Theory – an established qualitative method of categorizing empirically collected data, building a general theory to fit the data, and guiding data collection and analysis [12,13]. We initially constructed a maximum variety sample of 30 ophthalmologists to reflect a range of practitioner characteristics that could influence prescribing behaviours and opinions of the guideline [See Table 1]. In order to minimize regional bias, physicians were selected from both eastern and western Japan. Participants were recruited from different medical settings such as academic-affiliated hospitals and private practice or community hospitals, and represented a wide range of career stages to minimize bias derived from differential incentives for prescribing eye drops. We asked a research company to recruit participants via telephone from their database. A total number of 346 physicians were contacted.

In Japan, the majority of cataract patients are treated by ophthalmologists; however, some patients consult general practitioners (GP) for repeat prescriptions. This is particularly the case in rural areas where no specialist ophthalmology services are available. In this situation, patients initially consult urban ophthalmologists and then follow up with a local GP to receive their medication. Therefore, we interviewed an additional sample of three general practitioners. Another five ophthalmologists were interviewed to uphold the grounded theory principle of selection guided by the emerging analysis. The study protocol was approved by the Research Ethics Committee of the Kyoto University Graduate School of Medicine.

### Data collection and analysis

We conducted face-to-face, open-ended interviews at participants' clinics using a structured interview guide. Questions related to pirenoxine and glutathione eye drops included: frequency of prescription, perceptions about effectiveness, explanations to patients about their effectiveness, implementation of the related clinical guideline (see Appendix), and how the guidelines influenced their practices. One question related to opinions about the application of the EBM paradigm into health policy. When necessary, we added further questions to elucidate participants' perceptions of EBM.

Five trained interviewers conducted face-to-face interviews with participants and MS conducted eight interviews with the additional sample. Participants were encouraged to speak freely, discuss personally important issues, and to support their responses with examples from clinical practice, research, and/or policy when appropriate. All interviews were audio-taped and transcribed verbatim. We used constant comparative analysis to interpret the data [13]. MS and OT developed the structured interview guide, while MS and NK analyzed all interviews. All authors contributed to the analysis independently and discrepancies were discussed until final agreement. We scrutinized each interview line by line to identify main categories and concepts, which were then compared across scripts and with established concepts in published literature. Data collection and analyses were iterative, with new data used to assess the integrity of the developing analysis.

## Results

Physicians reported that, in general, patients anticipated the effectiveness of the eye drops and considered them to be the standard of treatment for cataracts. Physicians estimated prescription frequencies ranging from fewer than two in ten to "almost all" patients, many of which demanded such prescriptions. Approximately half of the participants conducted monthly follow-up visits with such patients, while the other half considered three-month intervals to be adequate.

### Opinions regarding the effectiveness of the eye drop

Most physicians reported never having witnessed objective effectiveness of cataract eye drops in their clinical practice. Only one ophthalmologist claimed that eye drops were definitely effective in approximately 20–30 percent of patients, recounting his experience with a patient who had been able to avoid surgery with these medications. Nevertheless, 23 physicians did not rule out the effectiveness of these drugs, citing cases in which symptoms did not progress when these medications were administered on a long-term basis, and that that patients were more willing to maintain contact with them in order to continue taking the drops.

The grounds for perceived "true effectiveness" were governmental approval of the drops, medical properties based on physiology, bench research or "first principles." The majority of participants believed that the lack of studies proving the effectiveness of the drops was not neccessarily proof of ineffectiveness. Three reasons were put forward for this position: 1) difficulty assessing the effectiveness of preventative medications using conventional epidemiological methods; 2) psychological benefits, which are not generally measured in clinical epidemiology; and 3) the lack of large clinical trials proving the ineffectiveness of the drugs.

Eight ophthalmologists refuted the effectiveness of such medications outright. However, such statements were primarily based on clinical experience rather than on "scientific evidence." GPs generally had no firm opinion regarding the effectiveness of the drops. Despite that GPs did not think that such medications had remarkable effects, they believed them to be clinically effective since they are widely used by experts and frequently requested by patients.

All but one physician agreed that eye drops are necessary to some patients for *psychological comfort*. Although some participants considered this effect to be spurious, they still argued that such placebo effects should be validated.

### Attitudes and behaviours in prescribing the eye drops

None of the participants adopted a clinical practice in which they refused to prescribe eye drops to patients. Physicians' prescription behaviours were broadly divided into four categories; (1) automatic or active prescription, (2) passive prescription, (3) prescription based on patient delegation, and (4) avoiding prescription.

Seventeen ophthalmologists automatically prescribed the eye drops. Eleven physicians reported passive prescription in response to patients' requests. They did not believe them to be clinically effective, and never recommended them out of their own volition. However, when patients requested these preparations, they prescribed them without discussion. GPs who had no opinion about the effectiveness of the drops also prescribed them automatically when requested. That is their role was merely to write a prescription based on a patient's request, rather than to make a formal prescribing decision. Since patients had already been prescribed the drug by an ophthalmologist, the GPs felt they were not in a position to challenge patients' preferences or beliefs, considering it safer to follow the ophthalmologist's advice. In this respect, GPs' attitudes toward prescription were passive, although their prescription behaviour was automatic.

Six physicians, five of whom practised in teaching hospitals, delegated the prescription decision to patients. These participants tended to vacillate in their opinions of the effectiveness of these drops; they did not observe remarkable clinical effects, but recognised that the preparations contain active ingredients and appreciated their psychological effects. Lacking a definitive opinion on the drugs' effectiveness, these participants preferred to provide information and allow their patients to make the ultimate decision.

All but two ophthalmologists stated that they usually discussed uncertainties or limitations regarding the effectiveness of the medications with their patients. Among participants who shared this perspective, however, explanations varied widely. While some affirmed the effectiveness, even if not emphasizing it, many subtly implied that the drops helped prevent the progression of cataracts. An ophthalmologist who believed the drops to be effective reported that he often encouraged patients to use them, citing cases in which the drug had dramatic effects. Another ophthalmologist, who automatically prescribed the drugs, encouraged patients to use them by displaying an image of the cornea and then advoctaing that they maintain control over the illness. Eight ophthalmologists reported trying not to use expressions that refute the effectiveness of the eye drops, regardless of their own beliefs. Four encouraged patients who eagerly used the drugs; their explanations included, "Your efforts will be paid off," or "At this point, we cannot say these eye drops are ineffective, but we may know in the future." Four physicians tried to dissuade patients from requesting these drugs by giving negative explanations of their effectiveness. As reported below, this approach was sometimes successful:

Ophthalmologist:*"For the last few years, I have usually said threateningly to a patient who requests these eye drops, 'You cannot discontinue them once you have started. Because the effectiveness is subtle, they lose their strength if used intermittently. Do you think you can continue to apply these drops several times a day, every day, from now on?' Putting it that way, patients in my neighbourhood are quick to understand and few still insist on the eye drop. But my colleagues tell me that such understanding patients are seldom encountered in their locality"*.

### Knowledge of and attitudes toward the paradigm of evidence-based medicine (EBM)

Twenty-eight participants reported being familiar with or had encountered the term "EBM." However, only seven agreed with the paradigm. The most difficult principles to support were "assessing treatment effectiveness based mainly on clinical research," "assessing effectiveness based on its magnitude," and "assessing treatment effectiveness in a patient group, rather than in individual patients." These 28 ophthalmologists reported that although the guidelines mention an absence of scientific evidence supporting drug effectiveness of eye drops, they believed that their effectiveness or ineffectiveness has yet to be proven. Opinions of physicians who supported the EBM paradigm were divided in terms of the drugs' effectiveness. Some believed that a lack of evidence proving effectiveness is equivalent to the drugs being ineffective, while others thought that their effectiveness has yet to be refuted.

When we asked participants whether they considered scientific evidence of effectiveness in decisions to prescribe a drug, 27 replied that this is not possible in the case of cataract eye drops, because there is currently no solid conclusion regarding drug effectiveness. Five also stated that as no alternative medications exist for cataracts, the only remaining option is to treat patients conservatively with the eye drops until surgery is necessary. They assumed that they had to provide something for patients who consulted them, regardless of scientific evidence.

Seven physicians reported feeling more comfortable using medications with proven effectiveness. However, they would still use the drugs even in the absence of such scientific evidence, particularly if only one treatment is available. Evidence of effectiveness was important only when needing to compare multiple treatment options.

One physician expressed concern that EBM methodology is not suitable to assess the "psychological effects" of a drug:

Ophthalmologist: *"I think the assessment of treatment effectiveness based on EBM fails to consider the view of patients diagnosed with cataract who feel tremendously insecure if no action (such as applying an eye drop) is performed. Although patients' anxieties may be considered to be unscientific in EBM, I think it's an important issue that clinicians cannot ignore."*

### Maintaining the physician-patient relationship

Prescribing the eye drops was acknowledged as one of the most important factors in maintaining the physician-patient relationship. Regardless of beliefs about their effectiveness, ophthalmologists agreed that the primary objective of the prescription was to satisfy patients who demand them. One ophthalmologist stated that patient satisfaction is one of the most important duties of a physician, and that drugs that do neither harm nor good are sometimes useful for that purpose. Ten ophthalmologists reported using the eye drop as a tool to maintain a good relationship with patients and to establish their reputation as a responsible physician.

Physicians noted that some patients consider it important to receive the standard of care for cataract treatment. Therefore, they believed that not prescribing the drug would lead to patient dissatisfaction and distrust. One ophthalmologist stated:

Ophthalmologist: "*I had an experience with one patient to whom I didn't prescribe the drug, after explaining that it's supposed to be ineffective. When his symptoms got worse, he came to me and said, 'I wonder if my cataract progressed because you didn't give me the drug.' He didn't appreciate my explanation that the effectiveness of these eye drops is uncertain or that progress of the illness mostly depends on characteristics of the individual patient. Consequently, I felt that I had to prescribe the drug*."

Another ophthalmologist explained that he worries that patients do not appreciate physicians who faithfully follow EBM guidelines and not prescribe the eye drops. Conversely, an ophthalmologist who described herself as a "low prescriber" reported experiencing little difficulty in convincing patients not to use the drops. Most patients were persuaded by her explanation that the eye drops are useless and did not further request the drug. However, she also reported prescribing the drugs on occasion to some patients who continued to demand them. She believed that persuaded patents did not visit other ophthalmologists in order to obtain the drugs, but rather consult her for routine check-ups every six months to one year. Another GP stated that successful interactions with patients are dependent upon a trusting relationship between patient and provider:

GP: *"Although I am not an expert, when I tell them that the effectiveness of these eye drops is uncertain, quite a few patients who trust me may take my advice seriously. If we doctors fully understand the paradigm of EBM, and clearly explain that the eye drop sometimes has side effects and is not very effective, we'll succeed in persuading patients. But at the same time, we must be accountable to them for the reasons why we have prescribed these drugs for so long."*

### Economic incentives of prescribing the eye drops

Two clinic-owning ophthalmologists who did not provide surgical treatment at their clinics reported that they would have financial difficulties if they did not prescribe these eye drops; however, they insisted that moneymaking was not the primary reason for this practice. One ophthalmologist who had recently purchased his clinic described the economic incentive in prescribing the eye drops.

Ophthalmologist: "*Before I owned my clinic, I seldom offered patients the eye drops, because I didn't want to provide medical care which isn't supported by scientific evidence. When patients demanded, I explained that they are ineffective, and prescribed them only when they still demanded. But now, I find myself giving the drugs to patients without discussion, but hinting, 'It's good for your eye.' I cannot help but attend to business"*.

We observed an association between physicians' affiliation and their attitudes toward prescription. Ophthalmologists who owned private clinics were more likely to prescribe the drugs automatically or actively, those in community hospitals were more likely to prescribe them passively, and those in teaching hospitals were more likely to delegate the decisions to the patients. However, five hospital-based physicians prescribed the drug automatically.

### Implementation of the guidelines and changes in clinical practice

Two ophthalmologists considered the guideline recommendations to be exceedingly vague. When we asked participants about guideline implementation, 23 physicians replied that they had already adopted the recommendation to "prescribe the drugs with adequate informed consent" prior to reading the guidelines. They had always explained the uncertain effectiveness of these drops to patients and prescribed them only to those who still requested them, thus, their prescription behaviours were not altered by the guidelines. However, when we asked participants what they actually say to patients during informed consent, 31 physicians replied that they usually touched upon the uncertainty of the drugs' effectiveness. Ten physicians considered a detailed explanation about evidence of effectiveness unnecessary because they believed that patients would neither want nor understand it:

Ophthalmologist: "*I don't tell them everything known in regard to evidence. Of course, if they had serious side effects or were proven to be completely ineffective, I would explain that. But for now, whether the drugs are ineffective has yet to be determined, and the drug is still approved by the government, I don't think we have to tell patients what is uncertain"*.

Six physicians interpreted the guidelines as instruction to not prescribe the drugs. However, these participants had adopted the guidelines into their practices prior to their issue. Two of them reported being relieved, since the guidelines' description of the drugs' effectiveness was consistent with their beliefs. However, one GP, who passively prescribed the eye drops, pointed out the complexities of informed consent procedures.

GP: "*The phrase 'with adequate informed consent,' which is used in many Japanese guidelines is very difficult to perform. Patients with cataract are usually elderly people who prefer paternalistic treatment and are willing to delegate decisions to physicians. On top of that, I'm afraid that patients in general don't understand why these eye drops, which had previously been judged effective by the government, are now judged ineffective by EBM*."

Fifteen physicians were questioned by patients about the effectiveness of the eye drops after encountering media coverage of the issue. Some discontinued prescribing the drugs at this point. An ophthalmologist who actively prescribed the drugs mentioned that his patient had reported believing the drops to be effective regardless of the press coverage. Consequently, physicians reported that only a few patients had discontinued the drugs.

### EBM and health policy

When we asked participants whether they thought the coverage of these eye drops by health insurance should be revoked based on "evidence" of their ineffectiveness, about one third disagreed with this statement. The primary reason for this was quite simple; they did not consider such drops to be futile and appreciated the advantages of using them in their practices, considering the absence of an alternative drug. Eleven ophthalmologists were concerned about having to explain to patients why they had prescribed the drugs for so long if they were determined to be ineffective and revoked by the government. Four physicians were confounded by the discrepancy between the "effectiveness" that was once stated by the government and then later assessed by EBM. They experienced embarrassment from the sudden change in assessment of drug effectiveness by the authority, as this resulted in the standard treatment they had performed in good faith being labelled as "scientifically ineffective."

Ophthalmologist: "*I have believed these drops to be effective for a long time. Therefore, I can't accept that they aren't effective now. I would have preferred that the government approved the drug with a more adequate review. Physicians as well as patients were shocked after using the eye drops over a long period. It's too late say to my patients, 'Recently they turned out to be ineffective.'*"

Ten physicians thought that approval should only be revoked if the eye drops were proved truly ineffective; however, they considered it too early to determine effectiveness based on such scarce data. These physicians stressed the necessity of conducting large clinical trials to investigate the "true effectiveness" of these drugs, and thereby obtain "consent" from all physicians to revoke approval. One ophthalmologist expressed her distrust of the government's attitude toward the approval and recent reassessment of the drug's effectiveness without scruples:

Ophthalmologist: "*Why did the government approve the drugs before they correctly evaluated their effectiveness? Although those who made the approval decision are to blame, the guideline was issued in such a manner that the government does not claim any responsibility; consequently patients blame physicians for this policy*."

## Discussion

This study aimed to identify how the practices of ophthalmologists and GPs are influenced by so-called "scientific evidence." We selected the recommendations regarding eye drop treatment (as contained in the Evidence-based Guidelines for Cataract Treatment) as a research topic for the following reasons. First, these medications are greatly accepted among the general public in Japan, recommended by many medical texts as the standard of care for cataracts, and are also covered by national health insurance. Second, physicians' perceptions of the effectiveness of these drugs and how evidence has changed these perceptions are not well known. Third, prescribing the drugs brings obvious economic benefits to providers under the fee-for-service payment system in Japan. Although the drugs themselves are inexpensive (around 200 yen ($1.90) for a 15 ml bottle), patients who consult doctors to refill prescriptions often concurrently receive routine medical check-ups, which elevates the total expenditure associated with the prescription of eye drops. Fourth, these drugs have considerable economic impact on Japan's healthcare system. The annual domestic sales are approximately 18 billion yen ($176 million) [14], and cataract-related outpatient care was estimated to cost the nation approximately 78 billion yen ($743 million) in 2002 [15]. Lastly, despite the guidelines, the government still approves the coverage of these drugs under the national health insurance plan.

Several studies have investigated the reasons why physicians sometimes provide treatment with marginal effectiveness. Butler et al. investigated the reasons why GPs prescribe antibiotics for a sore throat despite the lack of evidence supporting this practice. They concluded that although doctors know that antibiotics do not help most sore throat sufferers and find prescribing "against the evidence" uncomfortable, they try not to jeopardise relationships with patients over this issue [16]. They also argued that consistent evidence for a lack of effectiveness is unlikely to change physicians' prescribing practices. Conversely, Kumar et al. argued that GPs are mostly comfortable with their prescribing decisions; therefore, maintaining favourable physician-patient relations is not the primary objective of their prescribing practices [17]. However, in these particular studies, the influence of scientific evidence on changes in physicians' practices has never been compared with those of economic incentives, standard of care expectations of both physicians and patients, and authority approval.

### Rationale for prescribing

This study revealed that despite their extensive use, very few physicians prescribed the eye drops with expectations that any objective signs of clinical effectiveness would result. Nevertheless, few physicians agreed that the drugs lack effectiveness. On the surface, it seems paradoxical that physicians assert the effectiveness of the eye drops while simultaneously denouncing the objective effectiveness of these drugs. The best possible explanation for this contradiction is that effectiveness itself is not an essential factor in a physician's decision to write a prescription; for most physicians, the effectiveness of the eye drops did not necessarily need to bring remarkable clinical benefits to patients.

Physicians believed that prescribing eye drops was the easiest and most secure method of satisfying patients. When patients complained of decreased visual acuity and expected eye drops, many physicians felt uneasy sending them home empty-handed. Although physicians cited patients' requests as the main reason for prescribing the eye drops, they were not always concerned whether patients truly wanted them. Physicians seldom considered that prescribing might disadvantage patients; on the contrary, they were concerned that patients would not appreciate physicians who gave a complicated explanation of EBM instead of prescribing the eye drops. Therefore, even though physicians were not uncomfortable with providing treatment that is not supported by evidence, they did express discomfort with guidelines that contradict their actions.

The validity of the prescription, including the risks and benefits of daily use of the eye drops, was seldom discussed between physicians and patients. One possible reason for this is that physicians felt that doing so would be time consuming and unrewarding for the patient [16]. Japanese medical settings are particularly unfit for demanding encounters with patients. In fact, because patients have free access to all medical institutions, they often have to wait three hours for a three-minute consultation [18]. Another possible reason is that physicians do not have firm convictions about the ineffectiveness of the eye drops. Before the guidelines were issued, most physicians had prescribed the eye drops without giving much thought to their effectiveness, with the only criterion being permission from the government to cover the drugs under the national health insurance system. Although one physician suggested that patients are willing to take advice from a physician to avoid useless drugs, others believed that patients were more likely to be satisfied when they used the eye drops.

Physicians also acknowledged the various benefits of prescribing drugs that outweigh their uncertain effectiveness. These included enhancement of the doctor-patient relationship by improving patient satisfaction and psychological comfort, as well as believing that they were providing satisfactory medical treatment to patients. Economic benefit was also cited as a sensible reason for prescription. In the Japanese universal health insurance system, enrolment is mandatory and all reimbursements to providers are paid on a fee-for-service basis. Payments made by patients out-of-pocket are quite low since drugs are fully covered by the insurance. Therefore, physicians are free to make treatment decisions without worrying about the patient's ability to pay. A cultural aversion to invasive procedures may also explain why both physicians and patients prefer conservative treatment such as eye drops, rather than surgical treatment.

Some readers may be apprehensive about whether there is an excess of patients who request unnecessary medications. Our study did not clarify whether physicians or patients took the lead in establishing this pattern, nor could we find additional studies regarding patients' beliefs and attitudes toward eye drops Previous studies have revealed that physicians' perceptions of patient expectations are the strongest predictors of prescription decisions [16,19,20]. Britten and Ukoumunne found that physicians wrote a prescription to patients who seemed to want one even when they considered it to be clinically unnecessary [20]. In their study, 22% of prescriptions by GPs were not strictly indicated on medical grounds. Similarly, Scott et al have investigated the ways in which patients pressure physicians into prescribing antibiotics for respiratory infections. Their study found that, although physicians prescribed an antibiotic to roughly 68% of patients with an acute respiratory infection, approximately 80% of prescriptions were influenced by patient pressure and considered to be clinically unnecessary [21]. Our findings showed this same propensity for physicians to prescribe as a means to satisfy patient wishes. Interestingly enough, this contrast sharply with the common belief that Japanese physicians are paternalistic [22]. Our findings showed that patients' opinions were often more relevant than clinical necessity when prescribing eye drops. Such a strong appreciation of medication by both physicians and patients may originate from the tradition of ancient Japanese medicine, in which medication was the primary treatment and medical practitioners were often considered to be pharmacists [23].

Our study suggests the possibility of changing physicians' prescription behaviours through increasing patients' awareness of scientific evidence. Most guidelines in Japan are solely intended for physician use. When patients begin to appreciate physicians who make an effort to provide evidence-supported medical care, it is likely that physicians will change their prescription behaviours. Further approaches to clinical guidelines should focus on how to make clinical decisions with the active involvement of patients. Economic incentives for evidence-based medical care are also important in changing physician practices. Unfortunately, the current fee-for-service payment system brings obvious economic disadvantages to providers who limit the number of prescriptions.

We must consider what healthcare provides for patients; namely, does it only provide medical care supported by scientific evidence, or does it also provide psychological comfort to patients (rather difficult to assess by EBM)? Our study focused on physicians' prescribing behaviours related to drugs that lack evidence of effectiveness." Therefore, the findings may not be applicable to other drugs with considerable evidence of effectiveness; however, many drugs with marginal evidence of effectiveness are currently prescribed in Japan. Although our study does not answer the question of whether evidence-based healthcare and treatment is superior to treatment with marginal effects, both physicians and patients should consider what constitutes the integrity of medical care. Even though the prescription of drugs with marginal effects is a convenient tool for maintaining good physician-patient relations, such an easily obtainable prescription may compromise the quality of medical care in terms of practicality, utilization, and the balance between risks and benefits. Japan's health care system should be designed so that it does not conflict with quality medical care.

## Conclusion

Occasionally physicians prescribe drugs without expecting remarkable effectiveness. In such cases, evidence of effectiveness seldom changes their clinical practices or prescribing behaviours. Cataract eye drops are a typical illustration of this phenomenon. Many determinants influence physicians' prescribing behaviours. The effectiveness of a drug assessed by the EBM paradigm is only one such consideration. For physicians to change their practices according to evidence-based guidelines, they must recognize, and understand, and agree with the evidence, feel confident about their abilities, and overcome the inertia of previous practices [24]. The absence of any one of these components could prevent the evidence from taking root in physicians' clinical practices. It is proposed that they are more likely to alter such patterns when the change results in better patient outcomes. Unfortunately, discontinuing the prescription of these eye drops does not currently seem to be a desirable option for either physicians or patients. These findings support the hypothesis that replicated scientific evidence alone does not easily change physician behaviours [16], unless evidence-supported clinical practices are accepted by the general public and supported by health policies.

## Competing interests

This study was supported by Pfizer Health Research Foundation Grants. Although the Foundation is partially sponsored by Pfizer Japan Inc., the organization is a pubic-interest cooperation supervised by the Ministry of Health and Welfare Japan, and decisions on research funding are independent from the interests of the pharmaceutical company. None of the authors has ever received reimbursements, fees, funding, or salary from private companies or organizations which may in any way gain or lose financially from the publication of this manuscript.

## Authors' contributions

MS and YI conceived the study, and MS and OT developed the structured interview, while MS, NK and IT analyzed all interviews. All authors contributed to the analysis independently, and discrepancy between the analysis and results was discussed until final agreement.

## Appendix

Recommendation of the Evidence-based Clinical Guideline for Cataract Treatment:

Physicians can consider prescribing eye drops for patients with early stage senile cataract. Since there is no scientific evidence supporting the effectiveness of these drugs, it is desirable to give patients the drugs with adequate informed consent. (Grade of Recommendation: level C)

## Pre-publication history

The pre-publication history for this paper can be accessed here:



## Figures and Tables

**Table 1:** Characteristics of 30 ophthalmologists in the maximum variety sample

	No of ophthalmologists
Gender	
Male	24
Female	6
Site of practice	
Teaching hospital	9
Private practice	14
Community hospital	7
Area	
Western Japan	21
Eastern Japan	9
Age	
Younger than 40	11
40–49	13
Older than 50	7
